# Assessment of cephalometric parameters and correlation with the severity of the obstructive sleep apnea syndrome

**DOI:** 10.1186/s12967-024-05194-8

**Published:** 2024-04-22

**Authors:** Eugenio Garofalo, Giuseppe Neri, Lucilla Maria Perri, Nicola Lombardo, Giovanna Piazzetta, Alessandro Antonelli, Eugenio Biamonte, Vincenzo Bosco, Caterina Battaglia, Corrado Pelaia, Francesco Manti, Annalisa Pitino, Giovanni Tripepi, Andrea Bruni, Michele Morelli, Amerigo Giudice, Federico Longhini, The OSAS Study authors, Selene Barone, Antonio Caroleo, Angela Corea, Giusy Guzzi, Lucia Lentini, Sebastiano Macheda, Pietro Maglio, Helenia Mastrangelo, Alessandra Pasqua, Marianna Salviati, Marco Tescione

**Affiliations:** 1 Department of Medical and Surgical Sciences, Magna Graecia University, Viale Europe, 88100 Catanzaro, Italy; 2 Department of Health Sciences, School of Dentistry, Magna Graecia University, Catanzaro, Italy; 3 Department of Otolaryngology, “R. Dulbecco” University Hospital, Catanzaro, Italy; 4 Department of Anesthesia and Intensive Care, “R. Dulbecco” University Hospital, Catanzaro, Italy; 5 Department of Radiology, “R. Dulbecco” University Hospital, Catanzaro, Italy; 6 IFC-CNR, Rome, Italy; 7 IFC-CNR, Reggio Calabria, Italy; 8 Department of Obstetrics and Gynecology, “Annunziata” Hospital, Cosenza, Italy

## Abstract

**Background:**

In individuals diagnosed with obstructive sleep apnea syndrome (OSAS), variations in craniofacial structure have been inconsistently documented, showing differing degrees of alteration between obese and nonobese patients. In addition, sleep disturbance has also been shown to induce disequilibrium in this population of patients. This pilot observational study aimed to assess craniofacial values in obese and nonobese subpopulations of patients with OSAS and their correlation and association with the severity of OSAS. We also assessed whether OSAS patients are characterized by an impaired equilibrium in relation to and associated with the severity of OSAS.

**Methods:**

We included all consecutive adult patients with OSAS. Through cephalometry, we assessed the upper (UPa-UPp) and lower (LPa-LPp) pharynx diameters, superior anterior facial height (Sor-ANS), anterior facial height (ANS-Me), anterior vertical dimension (Sor-Me), posterior facial height (S-Go) and craniovertebral angle (CVA). Furthermore, we analyzed postural equilibrium through a stabilometric examination.

**Results:**

Forty consecutive OSAS patients (45% female with a mean age of 56 ± 8.2 years) were included. The subgroup of nonobese patients had a reduced *UPa-UPp (p* = 0.02). Cephalometric measurements were correlated with the severity of OSAS in nonobese patients, whereas only Sor-ANS was correlated with the severity of OSAS in the obese subpopulation. In the overall population, altered craniofacial values are associated with severe OSAS. Although there are differences in equilibrium between obese and nonobese OSAS patients, the stabilometric measurements were not correlated or associated with OSAS severity.

**Conclusion:**

Altered craniofacial values and compromised equilibrium in OSAS patients are linked to OSAS severity. Therefore, the management of OSAS should be tailored not only to weight management but also to craniofacial and postural rehabilitation to enhance patient outcomes.

**Supplementary Information:**

The online version contains supplementary material available at 10.1186/s12967-024-05194-8.

## Introduction

Obstructive Sleep Apnea Syndrome (OSAS) is a sleep disorder characterized by recurrent episodes of partial or complete obstruction of the upper airway during sleep, leading to disrupted breathing patterns, known as apnea or hypopnea, resulting in oxygen desaturations [1]. Various pathophysiological mechanisms contribute to OSAS, including impaired muscle responsiveness, the inability of the upper airway to dilate or stiffen in response to an increase in ventilatory drive, and notably, anatomical narrowing of the upper airways [2, 3]. The diagnosis of OSAS is established through cardiorespiratory monitoring and polysomnography [4]. Although some concerns exist [5], OSAS is classified for severity on the apnea–hypopnea index (AHI) as mild (AHI: 5–15), moderate (AHI: 15–30), or severe (AHI > 30) [6]. AHI is computed by summing the total number of apneas and hypopneas observed during sleep and dividing by the total sleep time in hours [6].

OSAS is associated with many diseases or disorders, such as cardiovascular diseases [7], metabolic disorders (e.g., diabetes) [8], gastrointestinal diseases (e.g., gastroesophageal reflux disease) [9], respiratory disorders (e.g., asthma) [10], emotional and psychological disorders [11] and increased mortality [12].

The gold standard for sleep apnea syndrome treatment is continuous positive airway pressure (CPAP), which consists of applying continuous pressure to the upper airways to keep them patent [13, 14]. In the case of moderate sleep apnea syndrome or failure of CPAP, a mandibular advancement device is a possible alternative therapy. It allows the jaw to advance, either fixed or progressive, increasing the diameter of the upper airways [15]. For obese patients, significant weight loss sometimes improves the symptoms associated with OSAS and reduces the correlated cardiovascular risk [16]. Maxillofacial surgery has proven to be quite effective [13]. In fact, OSAS patients are characterized by facial alterations that may benefit of surgical approaches, including improvements in associated symptoms like mandibular and cervical pain and bruxism [17, 18]. Therefore, the treatment of OSAS requires a holistic and multidisciplinary approach.

The influence of craniofacial morphology on the pathogenesis of OSAS is controversial [19]. Cephalometric evaluation has been used to determine the typical facial shape of OSAS patients [20]. The cervical, hyoid, and mandibular positions may affect the severity of OSAS [17, 18, 21]. Little is known about the use of the stabilizing platform used in OSAS, and it has not been widely applied [22–24]. In particular, stabilometric analysis is a method to assess postural control and balance, through the assessment of the center of gravity and the patient’s path length, under various conditions (opened or closed eyes and mouth) [22–24].

Despite the current extensive investigation of correlation between craniofacial alterations and the severity of OSAS [25–29], we designed this pilot observational study to assess craniofacial values obtained through cephalometric analysis and their correlation and association with the severity of OSAS (as defined by an AHI > 30). We further conducted a stabilometric analysis to assess whether OSAS severity could compromise equilibrium.

## Methods

This pilot observational study was conducted at the University Hospital “R. Dulbecco” of Catanzaro (Italy) from January to June 2023, following the STrengthening the Reporting of OBservational studies in Epidemiology (STROBE) guidelines for observational studies [30] and adhering to the ethical principles outlined in the Declaration of Helsinki and its subsequent amendments. Approval for the study was granted by the local Ethics Committee “Comitato Etico Sezione Area Centro—Regione Calabria” (Approval n. 372 on December 15th, 2022). Prior to participation, written informed consent was obtained from all enrolled patients. No modifications to the methods or study outcomes were introduced after the commencement of the study. Individual, deidentified datasets generated or analyzed during the study are available upon reasonable request from the corresponding author.

### Patients

We included all adult (i.e., ≥ 18 years/old) outpatients who visited the pneumology clinic and received a diagnosis of OSAS, established through type II polysomnography, as defined by the American Academy of Sleep Medicine guidelines [4]. Patients with one or more of the following criteria were excluded: (1) body mass index (BMI) > 37 kg/m^2^; (2) temporal-mandibular joint disorders [18], (3) fixed oral or mobile prosthesis; (4) previous radiant therapy in the head-district neck; (5) previous maxillofacial or upper airway surgery; (6) any smoking; (7) alcohol abuse; (8) use of sedative-hypnotic drugs (benzodiazepines, etc.), and/or comorbidities potentially affecting the equilibrium (for example vestibular disorders, cerebral stroke, neurodegenerative diseases, myopathies, arthritis, neuropathies). In addition, before study inclusion, all patients underwent dentistry and otorhinolaryngological examinations to exclude the presence of ankyloglossia and nasal obstruction and fibroscopy to assess the tonsillar and nasopharyngeal grading [31, 32].

The diagnosis of OSAS was obtained through cardiorespiratory monitoring and polysomnography (Somtè, Compumedics, Australia), as per guidelines [33]. The equipment comprises five cables for recording the electrocardiogram and heart rate (HR) via two bipolar leads, a nasal cannula to detect the flow-meter trace, a microphone for snoring recording, two piezoelectric belts for measuring thoraco-abdominal movements, a digital pulse oximeter for peripheral arterial oxyhemoglobin saturation (SpO_2_) measurement, and a gravity sensor for patient position tracking. The recordings were reviewed by the same operator and classified according to the American Academy of Sleep Medicine standards into obstructive, central, and/or mixed episodes, as well as apnoic and/or hypopnoic events [33]. Hypopnea was defined as oxygen desaturation by a 3% threshold. Sleep apnea was classified as central or obstructive based on the presence or absence of breathing effort. The oxygen desaturation index (ODI) assesses the frequency of desaturation episodes > 3% per hour of sleep, while time below 90% (TC90) calculates the fraction of saturation time < 90% [34].

### Data collection and analysis

For every patient anthropometric (BMI) and demographic (age and gender) and AHI data have been collected. Each patient underwent cephalometric analysis with OrisCeph software^®^ (Henry Schein ONE Srl, Cernusco sul Naviglio, Italy) and a stabilometric assessment (Lizard^®^, Lizardmed Srl, Monza, Italy).

**Fig. 1:**
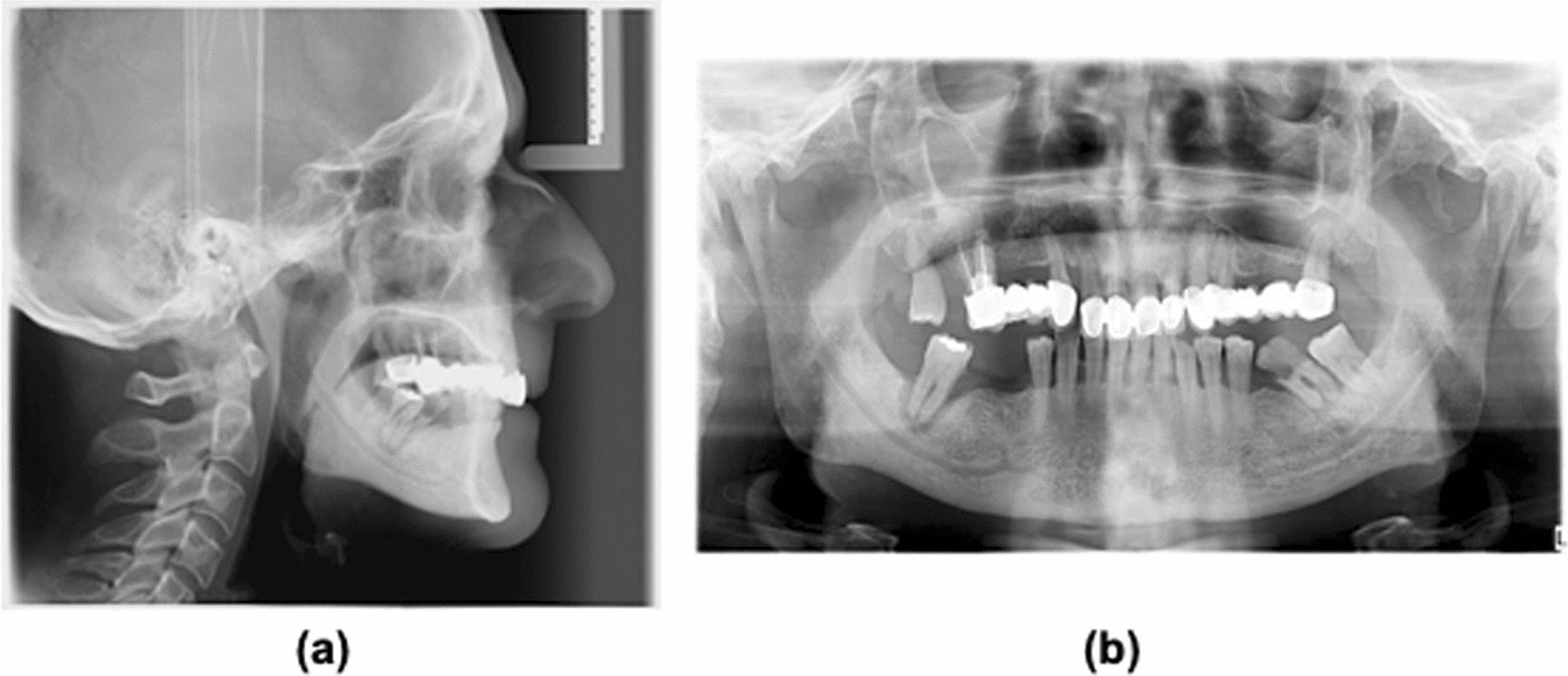
X–ray in lateral position (**a**) and Orthopantomography **(b)**

Cephalometry data were obtained by lateral-lateral skull teleradiography and orthopantomography for the analysis of the upper airways, skeletal bases, and cervical spine (Fig. 1).

**Fig. 2:**
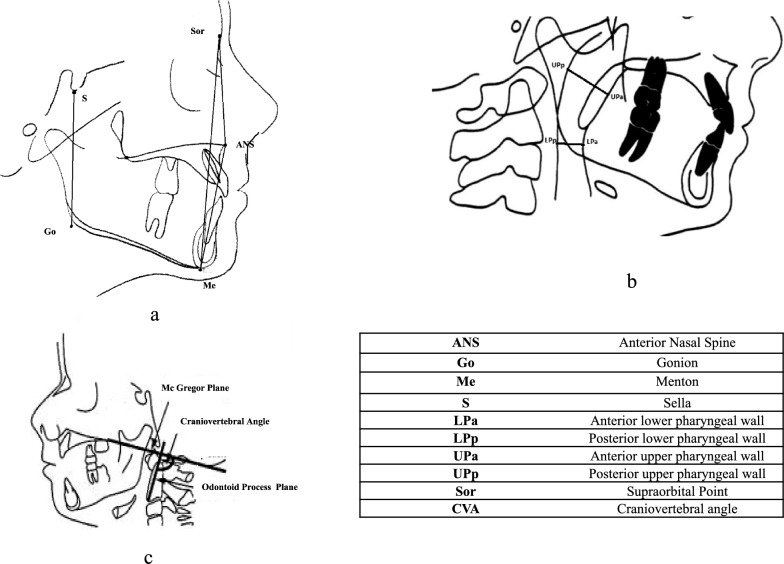
**a** measures of the distance between the differt facials points measured **b** diameter of upper and lower pharynx **c.**delimitation of craniovertebral angle

We assessed the following measurements, as depicted in Fig. 2:

- The antero-posterior diameter of the upper pharynx, measured between the posterior pharyngeal wall and the soft palatal tip, parallel to the palatal plane (UPa-UPp) [35];
- The antero-posterior diameter of the lower pharynx, measured between the posterior and anterior pharyngeal wall (base of tongue), along the mandibular plane (LPa-LPp) [35];
- The superior anterior facial height (Sor-ANS) distance between the supraorbital point (Sor) and the anterior nasal spine (ANS);
- The linear distance between the ANS and the menton (Me) (ANS-Me), representing the anterior facial height [29];
- The distance between the Sor and Me (Sor-Me), representing the anterior vertical dimension;
- The total posterior facial height (S-Go) assessed through the distance between the Sella (S) and the Gonion (Go) [27];
- The craniovertebral angle (CVA) is delineated by the McGregor plane (which extends from the base of the occipital bone to the posterior nasal spine) and the odontoid process (which spans from the apex of the odontoid process to the most anterior and inferior points of the C2 vertebral body) [36].

Stabilometric examination was performed using a static stabilometric platform (Lizard^®^, Lizardmed Srl, Monza, Italy) under three conditions: (1) open eyes (OE); (2) closed eyes (CE); and (3) open eyes with the mouth opened (MO). In all three settings, we assessed the surface area (SA) the patient used to search for the center of gravity and the patient's path length (PL). Finally, we calculated, under all conditions, the variance in the velocityas the ratio between the velocity of the center of gravity displacement and the number of measurements.

### Statistical analysis

Given the relatively small sample of patients, the data are expressed as the median (25th–75th interquartile range) and minimum to maximum (min–max) values. The data are presented for the overall population and for two subgroups of patients stratified according to BMI (< or ≥ 30 kg/m^2^). Continuous data were compared with the Mann‒Whitney U test. The correlation between the severity of OSAS and the severity of OSAS according to the cephalometric and stabilometric analysis was determined by Spearman rank correlation (ρ). Regression lines of rank-transformed data were fitted by BMI strata. Logistic regression analysis was performed to assess the association between cephalometric and stabilometric measurements and the presence of severe OSAS, as defined by an AHI > 30. According to the logistic regression model, the data are expressed as odds ratios (ORs) and 95% confidence intervals (CIs). P values < 0.05 were considered to indicate statistical significance in all tests. All analyses were performed using the R package (R Development Core Team).

## Results

We included 40 consecutive patients (45% female and 55% male). The average (SD) age was 56 ± 8.2 years. The mean (SD) AHI was 31.44 ± 13.8, and the mean (standard deviation) ODI was 32.16 ± 17.3. Twenty-three patients had a BMI < 30 kg/m^2^, whereas 17 patients had a BMI ≥ 30 kg/m2. None of the patients had ankyloglossia or nasal obstruction.

### Cephalometry

Cephalometric data from the overall population and from patients with a BMI < or ≥ 30 kg/m^2^ are reported in Table 1.

**Table 1:** Cephalometric measures in the overall population and stratified according to BMI

	Overall population (n = 40)	BMI < 30 kg/m^2^ (n = 23)	BMI ≥ 30 kg/m^2^ (n = 17)	P value
UPa-UPp-mm	12.8 [11–14.3] (6–17.6)	12.5 [10.6–13.8] (6–17.6)	14.2 [11.1–14.8] (10–17.4)	0.02
LPa-LPp-mm	11.15 [9.55–12.6] (7.1–17,7)	12.1 [8.9–12.6] (7.1–17.7)	11.1 [10.5–12.4] (8.3–17.5)	0.77
S-Go	61.75 [57.15–72.45] (26.7–80.07)	61.7 [57.1–72.2] (26.7–74.8)	61.9 [61.5–74.6] (33.9–80.07)	0.24
SOR-ANS-mm	54.4 [51.05–61.65] (24.3–65.4)	54.1 [52.3–56.8] (24.3–62.9)	60.3 [49.8–61.9] (38.5–65.4)	0.37
ANS-Me	57.3 [54.3–60.55] (27–77.9)	57.2 [54.2–60.1] (27–70.7)	60.4 [54.63–72.4] (38.4–77.9)	0.19
Grown SOR-ANS/ANS-Me	6.65 [1.8–8.7] (− 12–18.7)	7.13 [5.52] (− 4.6 to 14.1)	2.98 [7.77] (*− *12 to 18.7)	0.06
CVA	91.95 [83.4–101.7] (80.5–107.8)	94 [91.3–101.7] (80.5–103.9)	87.7 [83.3–103.7] (80.5–107.8)	0.66
SOR-Me	110.5 [106.24–120.25] (49.3–137.7)	110.3 [109.2–119.1] (49.3–125.5)	120.2 [100.2–134.91] (75.16–137.7)	0.20

Data are expressed as median [25th–75th interquartile range] and minimum to maximum (min–max)

P values are referred to comparisons between patients with BMI < 30 kg/m^2^ or ≥ 30 kg/m^2^

UPa-UPp, anterior–posterior diameter of the upper pharynx; LPa-LPp, anterior–posterior diameter of the lower pharynx; S, sella; Go, Gonion; SOR, supraorbital point; ANS, anterior nasal spine; Me, menton; CVA, CranioVertebral Angle

All measurements were similar between the two subpopulations stratified according to BMI (p > 0.05), except for the UPa–UPp (p = 0.02).

Table 2 shows the correlations between the measurements obtained from the cephalometric analysis and the presence of severe OSAS in the overall population and in the two subpopulations.

**Table 2:** Correlations between cephalometric measures and presence of severe OSAS in the overall population and stratified according to BMI

ρ	Overall population (n = 40)		BMI < 30 kg/m^2^ (n = 23)		BMI ≥ 30 kg/m^2^ (n = 17)	
	ρ	P value	ρ	P value	ρ	P value
BMI	0.23	0.15	0.28	0.19	− 0.08	0.77
ANS-Me	0.58	< 0.01	0.69	< 0.01	0.42	0.10
SOR-ANS-mm	0.64	< 0.01	0.79	< 0.01	0.58	0.02
S-Go	0.42	0.01	0.49	0.02	0.48	0.05
SOR-Me	0.61	< 0.01	0.81	< 0.01	0.40	0.11
CVA	− 0.27	0.09	− 0.28	0.20	− 0.24	0.36
UPa-UPp-mm	0.03	0.86	0.00	0.99	0.11	0.66

BMI, Body Mass Index; ANS, anterior nasal spine; Me, menton; SOR, supraorbital point; S, sella; Go, Gonion; CVA, CranioVertebral Angle; UPa-UPp, anterior–posterior diameter of the upper pharynx

In the whole study sample, significant correlations were found between the ANS-Me, Sor-ANS, S-Go, and Sor-Me scores and severe OSAS. These findings were further confirmed in patients with a BMI < 30 kg/m^2^. In contrast, in patients with a BMI ≥ 30 kg/m^2^, we found a significant correlation between Sor-ANS and severe OSAS (Table 2).

**Fig. 3:**
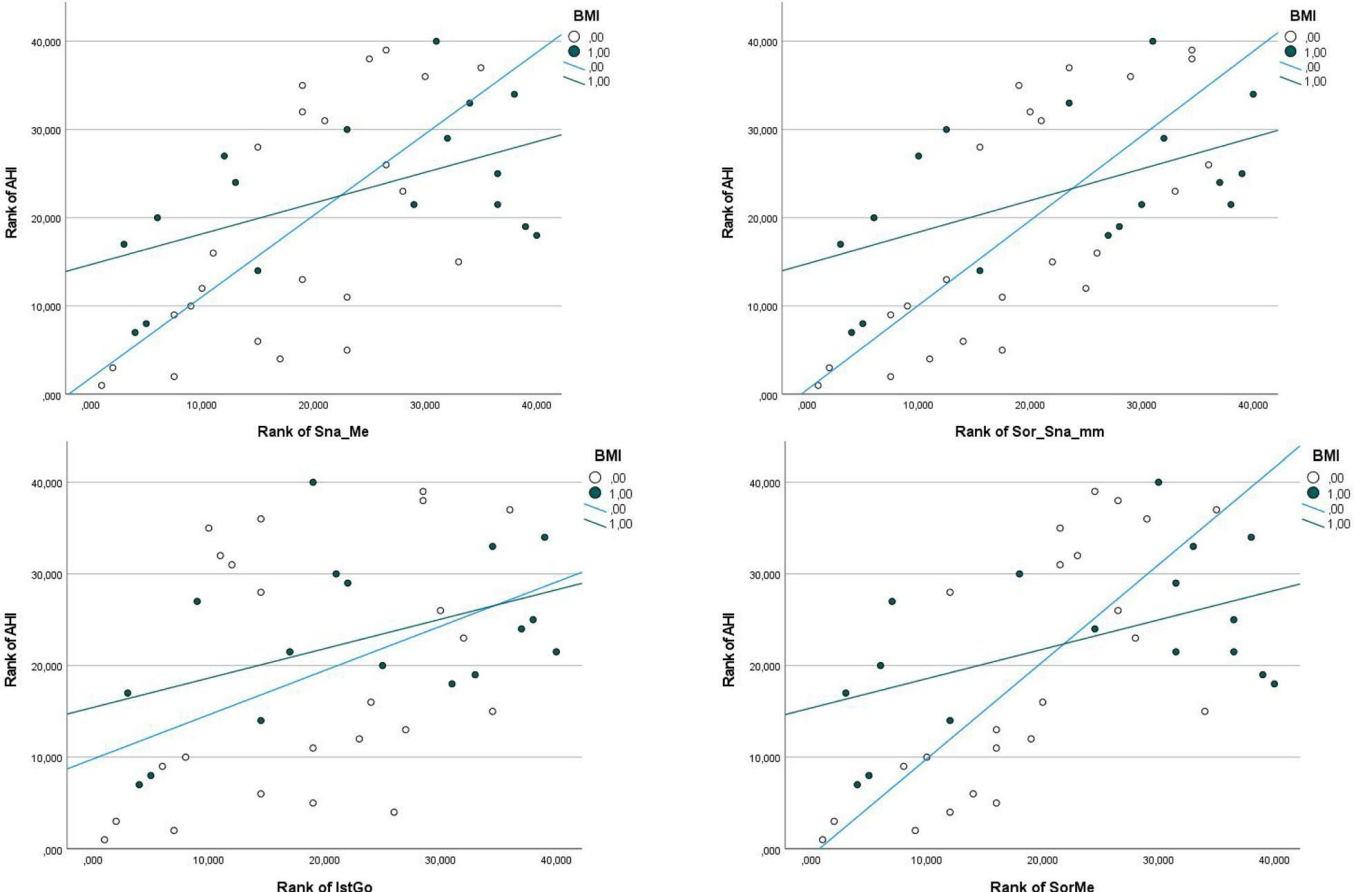
Regression lines between the AHI and the ANS-Me, Sor-ANS-mm, S-Go, and Sor-Me scores in patients with a BMI < 30 kg/m^2^ (hollow circles) and a BMI ≥ 30 kg/m^2^ (full circles)

The association between these variables and the AHI was stronger (i.e., greater slope) in patients with a BMI < 30 kg/m^2^ than in those with a BMI ≥ 30 kg/m^2^, as depicted in Fig. 3.

By chance, 20 patients had an AHI ≤ 30 (range 5 to 30), and 20 patients had an AHI > 30 (range 30.6 to59.8). As shown in Table 3, univariate logistic regression analyses demonstrated that ANS-Me, Sor-ANS-mm, S-Go, and Sor-Me were associated with a greater odds ratio for severe OSAS in the overall population. We also performed an analysis of the subpopulation of patients stratified according to BMI.

**Table 3:** Univariate logistic regressions of the presence of severe OSAS according to cephalometric data in the whole sample and in patients stratified by BMI

	Overall population (n = 40)		BMI < 30 kg/m^2^ (n = 23)		BMI ≥ 30 kg/m^2^ (n = 17)	
	OR (95% CI)	P value	OR (95% CI)	P value	OR (95% CI)	P value
ANS-Me	1.10 (1.01–1.19)	0.02	1.21 (0.94–1.56)	0.14	1.07 (0.98–1.17)	0.13
SOR-ANS-mm	1.30 (1.09–1.56)	< 0.01	1.60 (1.02–2.49)	0.04	1.20 (1.01–1.43)	0.04
S-Go	1.10 (1.01–1.16)	0.02	1.08 (0.97–1.21)	0.15	1.09 (0.99–1.19)	0.07
SOR-Me	1.10 (1.01–1.15)	0.02	1.23 (1.02–1.5)	0.03	1.05 (1.00–1.12)	0.07

ANS, anterior nasal spine; Me, menton; SOR, supraorbital point; S, sella; Go, Gonion; OR, odds ratio; 95% CI, 95% Confidence Interval

In patients with a BMI < 30 kg/m^2^, Sor-ANS and Sor-Me were associated with increased ORs for severe OSAS, whereas in patients with a BMI ≥ 30 kg/m^2^, only Sor-ANS was associated with severe OSAS.

### Stabilometric examination

Table 4 shows the stabilometric measurements assessed in the overall population and in the subgroup of patients stratified by BMI under different examination conditions (eyes open, eyes close and teeth contact).

**Table 4:** Stabilometric measurements in the overall population and stratified according to BMI

	Overall population (n = 40)	BMI < 30 kg/m^2^ (n = 23)	BMI ≥ 30 kg/m^2^ (n = 17)	*P value*
SA-OE mm^2^	69.88 [57.08–94.45] (25.94–315.57)	58.2 [41.27–92.1] (25.94–118.6)	79.32 [62.2–100] (55.13–315.57)	0.048
PL-OE mm	314.45 [290–381] (216.73–606.5)	291 [257.4–324.4] (216.73–424.62)	324.4 [308.78–381.1] (275.05–606.5)	0.015
SA-CE mm^2^	126.55 [76.75 217.77] (28.4–831.7)	77.2 [53.24–196.82] (28.4–357.46)	199.76 [126.5–278.7] (101.74–831.7)	0.001
PL-CE mm	495.05 [354.7–639.35] (229.6–979.63)	354.8 [280.5–546.14] (229.6–694.07)	546.12 [495.04–643.53] (409.1–979.63)	0.003
SA-MO mm^2^	69.33 [54.26–91.12] (25.87–689.72)	54.27 [35.2–87] (25.87–186.66)	83.74 [69.3–91.13] (59.35–689.72)	0.012
PL-MO mm	331.54 [286.5–377.28] (201.74–878)	287 [233.7–352.75] (201.74–493.76)	343.73 [331.53–380.55] (310.92–878)	0.004
Velocity variance	28 [16–34] (7–62)	19 [10–31] (7–54)	30 [26–41] (20–62)	0.008

Data are expressed as median [interquartile range] e (Min–Max). SA, surface area; PL, Path length; OE, opened eyes; CE, closed eyes; MO, mouth opened

Compared to patients with a BMI < 30 kg/m^2^, those with a BMI ≥ 30 kg/m^2^ were characterized by higher values of SA, PL and velocity variance in all the examined conditions.

Notably, we did not find any correlation between the stabilometric measurements and the AHI, either in the overall population or in the subgroup of patients stratified according to BMI. An additional file shows this in more detail (see Additional file 1).

## Discussion

In this pilot observational study, we found that obese and nonobese OSAS patients have different craniofacial morphologies that are correlated with the AHI and are associated with severe OSAS. In addition, although obese OSAS patients were characterized by a different equilibrium as assessed by a stabilometric assessment, we could not find any correlation or association with the severity of OSAS.

Research on cephalometric predictors in patients with OSAS is a cornerstone of sleep medicine [37, 38], as is the investigation of early predictive factors such as craniofacial morphology in children [39]. In OSAS, upper airway obstruction can originate from various sources, including soft tissue collapse, such as the soft palate, or alterations in the position or dimensions of structures such as the tongue, maxilla, or mandible [40–43]. Upper airway obstruction can lead also to intermittent hypoxia, that has systemic effects in OSAS individuals [44]. The evaluation of craniofacial soft tissue is also crucial. For instance, Lee et al. reported that OSAS patients often exhibit broader and flatter mid- and lower-thirds of the face, accompanied by reduced maxillary and mandibular lengths [45]. Similarly, Tyan et al. identified significant correlations between craniofacial measurements and the severity of OSAS [46].

Our findings are in line with previous data reported by other studies. The evidence that OSAS patients are characterized by a different craniofacial morphology has also been described by Akpinar et al.; these authors reported that nonobese OSAS patients have smaller posterior airway spaces and different craniofacial morphologies than do habitual snorers (non-OSAS) and controls [19]. In fact, OSAS patients are characterized by mandibular micrognathias and retrognathia [47] and the retroposition of the maxilla and mandible [48–50]. OSAS are also characterized by a reduction in the posterior airway space or a multilevel obstruction of the airways [51, 52]. Indeed, systematic reviews have recently reported that a reduced pharyngeal airway space and inferiorly placed hyoid bone are the cephalometric parameters most strongly associated with the severity of OSAS [53, 54].

We herein reported that in nonobese OSAS patients, cephalometric measures were moderately correlated with the severity of OSAS; in addition, both the Sor-ANS and Sor-Me distances were associated with severe OSAS in nonobese patients, whereas only the Sor-ANS distance was associated with OSAS severity in obese patients. These findings may be explained by different underlying mechanisms in that subpopulation of patients. In fact, it may be postulated that in subjects with a BMI < 30 kg/m^2^, the severity of OSAS is mainly linked to alterations in skull facial dynamics. In these subjects, obesity likely plays a marginal role in the etiopathogenesis of the disease. Conversely, individuals with first-degree obesity develop OSAS for reasons binding mainly to obesity, which predisposes them to alveolar hypoventilation and airway collapse during sleep. In support of this hypothesis, Tangugsorn et al. also assessed differences in the cervicocraniofacial skeleton between obese and nonobese OSAS patients [55]. Researchers have found that among nonobese individuals with OSAS, anatomical irregularities are primarily limited to the skeletal structures of the cervical and craniofacial regions. In contrast, obese OSAS patients exhibit greater abnormalities in the soft tissue morphology of the upper airway, head posture, and position of the hyoid bone [55]. We also reported a certain correlation between the cephalometric assessment and the AHI (see Table 2).

Patients with OSAS exhibit sleep fragmentation and deprivation, which are suggested to be underlying factors contributing to impairments across various systems, including motor coordination [56–58]. The equilibrium is upheld through the ongoing and efficient integration of vestibular, visual, and proprioceptive inputs within the central nervous system [59]. These sensory data are consolidated and processed in the cerebellum, facilitating the stabilization and upkeep of the body's center of gravity through coordinated postural muscle contractions [60]. In this regard, OSAS patients were shown to be affected by a significant impairment in the vestibulo-ocular and sacculocollic reflexes and posturographic parameters [60–62]. It has also been suggested that these alterations may result from a chronic hypoxemic state that leads to a progressive reduction in vestibular function, generating disequilibrium [62, 63]. Consistent with a previous study [22], we observed an increase in the area and velocity variance with eyes closed and teeth in contact, whereas the area was reduced under conditions with eyes open and teeth in contact, indicating strong muscle rigidity and a continuous search for the center of gravity, supported by the abnormal increase in the length of the trace (the number of oscillations the subject makes to find the center of gravity). Interestingly, it has also been demonstrated that daytime postural stability is influenced by and associated with nocturnal breathing disorders [56]. In our investigation, however, we found neither a correlation between postural disorders and the AHI nor an association between measurements from stabilometric examination and the presence of severe OSAS.

Our study has several limitations. First, given the study aim, we were unable to compute a precise sample size. Although other studies have recruited larger numbers of patients than ours [48], here, we report the findings of a pilot observational study with a total sample (40 patients) similar to other studies [49, 50] or to their OSAS subpopulation [19]. In addition, we conducted nonparametric statistical analysis to be more conservative in the results [64]. Second, our findings may contrast with some literature data; this may also be due to an underpowered sample of patients. Third, given the observational nature of the study, the investigation may be affected by selection bias, and the findings may not be representative of a broader population; in addition, there could be several confounding variables [65]. This bias is dampened in our study by the enrollment of all consecutive OSAS patients, with no patients refusing to participate. Finally, while the measurements are standardized, it's important to note that these results are from a single-center study. Further investigations conducted at multiple centers are necessary to validate and generalize the findings.

## Conclusions

OSAS patients have altered craniofacial values, which are also related to the severity of OSAS according to the presence of obesity, although they are not the only determinants of severe OSAS. In addition, these patients have a compromised equilibrium unrelated to obesity and not associated with disease severity. Further and larger studies are required to confirm our findings.

### Supplementary Information

**Additional file 1: Table S1.** Correlations between stabilometric measures and presence of severe OSAS in the overall population and stratified according to BMI. **Table S2.** Univariate logistic regressions of the presence of severe OSAS according to stabilometric data in the whole sample and in patients stratified by BMI.

## Data Availability

The data that support the findings of this study are available upon request from the corresponding author. The data are not publicly available because of privacy or ethical restrictions.
