# Supplementary material for: Assessment of cephalometric parameters and correlation with the severity of the obstructive sleep apnea syndrome
Source: J Transl Med. 2024 Apr 22;22:377. doi: 10.1186/s12967-024-05194-8 (PMC11036665; doi:10.1186/s12967-024-05194-8)
Supplement: Supplementary file 1 — Additional file 1: Table S1. Correlations between stabilometric measures and presence of severe OSAS in the overall population and stratified according to BMI. Table S2. Univariate logistic regressions of the presence of severe OSAS according to stabilometric data in the whole sample and in patients stratified by BMI. [file 12967_2024_5194_MOESM1_ESM.docx]

ASSESSMENT OF CEPHALOMETRIC PARAMETERS AND CORRELATION WITH THE SEVERITY OF THE OBSTRUCTIVE SLEEP APNEA SYNDROME

Eugenio Garofalo, Giuseppe Neri, Lucilla Maria Perri, Nicola Lombardo, Giovanna Piazzetta, Alessandro Antonelli, Eugenio Biamonte, Vincenzo Bosco, Caterina Battaglia, Corrado Pelaia, Francesco Manti, Annalisa Pitino, Giovanni Tripepi, Andrea Bruni, Michele Morelli, Amerigo Giudice, Federico Longhini and the OSAS Study authors

- **Table S1. Correlations between stabilometric measures and presence of severe OSAS in the overall population and stratified according to BMI.**
- **Table S2. Univariate logistic regressions of the presence of severe OSAS according to stabilometric data in the whole sample and in patients stratified by BMI.**

**Table S1. Correlations between stabilometric measures and presence of severe OSAS in the overall population and stratified according to BMI.**

|  | **Overall population**  **(n=40)** | | **BMI <30** **kg/m^2^**  **(n=23)** | | **BMI ≥30 kg/m^2^**  **(n=17)** | |  |
| --- | --- | --- | --- | --- | --- | --- | --- |
|  | **ρ** | **P value** | **ρ** | **P value** | **ρ** | **P value** | |
| **SAOE mm^2^** | -0,11 | 0,48 | *-0,18* | *0,41* | -0,07 | 0,80 | |
| **PLOE mm** | -0,01 | 0,93 | *-0,10* | *0,68* | 0,16 | 0,54 | |
| **SACE mm^2^** | 0,04 | 0,76 | *-0,03* | *0,90* | 0,21 | 0,42 | |
| **PLCE mm** | 0,04 | 0,80 | *-0,04* | *0,86* | 0,24 | 0,35 | |
| **SAMO mm^2^** | -0,13 | 0,40 | *-0,16* | *0,46* | -0,01 | 0,70 | |
| **PLMO mm** | -0,09 | 0,60 | *-0,08* | *0,70* | -0,10 | 0,68 | |
| ***Velocity variance*** | 0,07 | 0,65 | *-0,15* | *0,48* | 0,48 | 0,05 | |

**Table S2. Univariate logistic regressions of the presence of severe OSAS according to stabilometric data in the whole sample and in patients stratified by BMI.**

|  | **Overall population**  **(n=40)** | | **BMI <30** **kg/m^2^**  **(n=23)** | | **BMI ≥30 kg/m^2^**  **(n=17)** | |
| --- | --- | --- | --- | --- | --- | --- |
|  | **OR (95% CI)** | **P value** | **OR (95% CI)** | **P value** | **OR (95% CI)** | **P value** |
| **SAOE mm^2^** | 0.99 (0.97-1.00) | 0.16 | *0,98 (0.95-1.02)* | *0,32* | 0,98 (0.96-1.01) | 0,15 |
| **PLOE mm** | 1.00 (0.99-1.01) | 0.52 | *1,00 (0.98 – 1.01)* | *0,72* | 0,99 (0.98 – 1.01) | 0,27 |
| **SACE mm^2^** | 1.00 (0.99 – 1.00) | 0.30 | *0,99 (1.00 – 0.22)* | *0,22* | 1,00 (0.99 – 1.00) | 0,28 |
| **PLCE mm** | 1.00 (1.00-1.00) | 0.56 | *1,00 (1.00 – 0.35)* | *0,35* | 1,00 (0.99- 1.00) | 0,40 |
| **SAMO mm^2^** | 0.99 (0.97 – 1.01) | 0.28 | *0,98 (1.01 – 0.19)* | *0,19* | 0,99 (0.98 – 1.01) | 0,47 |
| **PLMO mm** | 1.00 (0.99 – 1.00) | 0.34 | *1,00 (0.98 – 1.01)* | *0,34* | 0,99 (0.98 – 1.01) | 0,40 |
| ***Velocity variance*** | 1.02 (0.98 – 1.06) | 0.34 | *0,96 (0.9 – 1.03)* | *0,28* | 1,19 (0.94 – 1.51) | 0,16 |

OR, odds ratio; 95% CI, 95% Confidence Interval.
